# A Computational Toxicology Approach to Screen the Hepatotoxic Ingredients in Traditional Chinese Medicines: *Polygonum multiflorum* Thunb as a Case Study

**DOI:** 10.3390/biom9100577

**Published:** 2019-10-07

**Authors:** Shuaibing He, Xuelian Zhang, Shan Lu, Ting Zhu, Guibo Sun, Xiaobo Sun

**Affiliations:** 1 Beijing Key Laboratory of Innovative Drug Discovery of Traditional Chinese Medicine (Natural Medicine) and Translational Medicine, Institute of Medicinal Plant Development, Peking Union Medical College and Chinese Academy of Medical Sciences, Beijing 100193, China; wenyuxuan2530@139.com (S.H.); 13022517967@163.com (X.Z.); lushanluna@163.com (S.L.); linlang0402@foxmail.com (T.Z.); 2 Key Laboratory of Bioactive Substances and Resource Utilization of Chinese Herbal Medicine, Ministry of Education, Beijing 100193, China; 3 Key Laboratory of Efficacy Evaluation of Chinese Medicine against Glycolipid Metabolic Disorders, State Administration of Traditional Chinese Medicine, Beijing 100193, China; 4 Key Laboratory of new drug discovery based on Classic Chinese medicine prescription, Chinese Academy of Medical Sciences, Beijing 100193, China; 5 Zhongguancun Open Laboratory of the Research and Development of Natural Medicine and Health Products, Institute of Medicinal Plant Development, Chinese Academy of Medical Sciences & Peking Union Medical College, Beijing 100193, China

**Keywords:** Traditional Chinese Medicines, TCMs, drug-induced liver injury, DILI, computational toxicology, hepatotoxicity, *Polygonum multiflorum* Thunb

## Abstract

In recent years, liver injury induced by Traditional Chinese Medicines (TCMs) has gained increasing attention worldwide. Assessing the hepatotoxicity of compounds in TCMs is essential and inevitable for both doctors and regulatory agencies. However, there has been no effective method to screen the hepatotoxic ingredients in TCMs available until now. In the present study, we initially built a large scale dataset of drug-induced liver injuries (DILIs). Then, 13 types of molecular fingerprints/descriptors and eight machine learning algorithms were utilized to develop single classifiers for DILI, which resulted in 5416 single classifiers. Next, the NaiveBayes algorithm was adopted to integrate the best single classifier of each machine learning algorithm, by which we attempted to build a combined classifier. The accuracy, sensitivity, specificity, and area under the curve of the combined classifier were 72.798, 0.732, 0.724, and 0.793, respectively. Compared to several prior studies, the combined classifier provided better performance both in cross validation and external validation. In our prior study, we developed a herb-hepatotoxic ingredient network and a herb-induced liver injury (HILI) dataset based on pre-clinical evidence published in the scientific literature. Herein, by combining that and the combined classifier developed in this work, we proposed the first instance of a computational toxicology to screen the hepatotoxic ingredients in TCMs. Then *Polygonum multiflorum* Thunb (PmT) was used as a case to investigate the reliability of the approach proposed. Consequently, a total of 25 ingredients in PmT were identified as hepatotoxicants. The results were highly consistent with records in the literature, indicating that our computational toxicology approach is reliable and effective for the screening of hepatotoxic ingredients in Pmt. The combined classifier developed in this work can be used to assess the hepatotoxic risk of both natural compounds and synthetic drugs. The computational toxicology approach presented in this work will assist with screening the hepatotoxic ingredients in TCMs, which will further lay the foundation for exploring the hepatotoxic mechanisms of TCMs. In addition, the method proposed in this work can be applied to research focused on other adverse effects of TCMs/synthetic drugs.

## 1. Introduction

For thousands of years, Traditional Chinese Medicines (TCMs) have been widely consumed in many Asian countries. As natural products, TCMs were generally considered to be green and harmless [1]. However, actually, as all drugs do, TCMs also possess a series of side effects. The hepatotoxicity [2], nephrotoxicity [3], cardiotoxicity [4], neurotoxicity [5], carcinogenicity [6], and some other toxicities of TCMs have been reported in the literature. As one of the major concerns of TCMs, hepatotoxicity has gained more and more attention recently. Currently, hundreds of TCMs or their extracts have been reported to possess potential hepatotoxicity [7,8,9]. Several special databases focused on TCM-induced liver injuries also have been developed, such as Hepatox (http://www.hepatox.org) and HDS hepatotoxicity databases [10]. Additionally, in recent studies focused on the etiology of drug-induced liver injury (DILI) in several Asian countries, TCMs were found to be the leading induction factors of DILI [11,12]. TCMs with hepatotoxicity always resulted in severe clinical adverse events, such as liver fibrosis, hepatitis, liver failure, and even death [13]. Therefore, studies focused on the hepatotoxicity of TCMs are urgent and imperative.

The identification of hepatotoxic ingredients is always the first step to exploring the hepatotoxicity of TCMs. Unlike the synthetic drugs, which generally produce their efficacy based on the theory of one drug and one target, the efficacy/toxicity of TCMs relies on the comprehensive effects of multiple ingredients, multiple targets, and multiple pathways [14]. The molecular basis of a TCM is not a single chemical entity but the combination of many chemical ingredients. Therefore, it is difficult to unveil the mechanisms of TCMs solely based on the reductionism research strategies of Western medicine [15]. Network pharmacology, a burgeoning new field, analyzes the mechanisms of drugs by integrating the complex interactions among drugs, genes, diseases, and any other relevant entities in biological systems comprehensively. The basic theory of network pharmacology is in accordance with the holistic idea of TCMs [16].

During the last decade, network pharmacology has been applied to identify the active/toxic ingredients of many TCMs [17,18,19]. Recently, taking *Polygonum multiflorum* Thunb (PmT) as a case, Wang et al. proposed a pathway-based systems toxicology approach to understand TCM-induced liver injury [20]. As a result, a total of 54 compounds were found to be associated with the hepatotoxicity of PmT. The top seven compounds consisted of luteolin, kaempferol, gallic acid, resveratrol, apigenin, quercetin, and emodin. The hepatotoxicity of emodin has been well documented in many studies [21,22,23,24,25]. Luteolin was reported to cause cytotoxicity in primary rat hepatocytes at dosages of 50 μM or lower levels of concentration [26]. Apigenin was found to can significantly increase the accumulation of lipid droplets and cause fatty liver disease [27]. However, for the other four compounds, no direct evidence focused on their hepatotoxicity was retrieved. Inversely, all of those four compounds were reported to be promising hepatoprotectors [28,29,30,31,32]. In another study, a similar phenomenon was observed. Based on the network pharmacology framework, the authors attempted to identify the potential hepatotoxic ingredients in Xiao-Chai-Hu-Tang. As a result, kaempferol and thymol exhibited the largest number of hepatotoxic targets connections [33]. However, neither clinical cases nor scientific reports about the hepatotoxicity of those two compounds were available. In fact, both kaempferol and thymol could significantly attenuate the liver injury induced by several hepatotoxicants [32,34]. In the two representative cases mentioned above, one can easily find that those potential hepatotoxic compounds identified solely based on network pharmacology were the mixtures of beneficial and toxic ingredients. Therefore, to uncover the real hepatotoxic ingredients in TCMs, research focused on differentiating hepatotoxicants and non-hepatotoxicants is required.

Quantitative structure–activity relationship (QSAR) is aimed at correlating structure with activity [35]. In recent years, it has been widely applied to assess the hepatotoxic risks of the synthetic drugs [36,37,38]. However, QSAR research focused on evaluating the hepatotoxicity of TCMs is very rare. Huang et al. [39], Shi et al. [40], Liu et al. [41], and Wu et al. [42] have attempted to develop QSAR models to evaluate the hepatotoxic risks of ingredients from TCMs. However, all of those models were built solely based on the synthetic drugs. As we all know, the chemical environment of natural products is quite different from that of the synthetic drugs [43,44]. Therefore, in silico models developed solely based on the synthetic drugs are always not applicable to natural products. Based on the Liver Toxicity Knowledge Base, Zhao et al. [45] and Ye et al. [46] have attempted to develop QSAR models to evaluate the hepatotoxic risks of ingredients from TCMs. Although the training sets of those two studies were relatively small (*n* ≤ 350), they still indicated that QSAR models developed based on both TCMs and the synthetic drugs outperformed those only relying on the synthetic drugs.

In our previous studies, we collected two hepatotoxic datasets, among which, one was specially focused on herb-induced liver injury (HILI) [47,48]. In the current study, by integrating those two datasets and Comparative Toxicogenomics Database (CTD), we built a novel hepatotoxic dataset. Then, we attempted to develop QSAR models to predict the hepatotoxic risks of compounds by incorporating the use of 13 types of molecular fingerprints/descriptors and eight machine learning algorithms (NaiveBayes, LibSVM, IBK, KStar, AdaboostM1, Bagging, J48, and RandomForest). The recursive feature elimination (RFE) method was utilized to identify the optimal feature subset of each machine learning algorithm. As a result, the best model of each machine learning algorithm was attained. We then used the NaiveBayes algorithm to develop a combined classifier based on the eight best single classifiers. By integrating the three external validation sets collected by Ai et al. [37], Zhang et al. [49], and Kotsampasakou et al. [38], an integrated external validation set was acquired and utilized to test the reliability of the combined classifier. In addition, comparisons between the combined classifier and prior studies were conducted against the three external validation sets separately. Finally, taking PmT as a case, we proposed a computational toxicology approach to screen the hepatotoxic ingredients in TCMs by combining the combined classifier constructed in this work and the herb-hepatotoxic ingredient network and HILI dataset published in our prior studies [47,48].

## 2. Materials and Methods

### 2.1. Data Set

As a robust, publicly available database, CTD is focused on understanding the interaction between the environmental exposures and human health [50]. We searched CTD with the term, “Chemical and Drug Induced Liver Injury.” Only those hepatotoxic compounds curated by experts were preserved. As a result, a total of 1009 hepatotoxic compounds were collected. Then, the data preparation method used in our prior study was adopted to unify the structures of compounds collected from CTD [47]. In our prior studies, two hepatotoxic datasets were compiled by retrieving scientific publications comprehensively [47,48]. Of note, one of the datasets is specially focused on HILI, including 584 non-hepatotoxic ingredients and 296 hepatotoxic ingredients. We integrated those two datasets and the CTD dataset, deleting duplicates and compounds that appeared in the external validation sets. As a result, a novel dataset of DILI, including 1049 hepatotoxic compounds and 1142 non-hepatotoxic compounds, was built. This dataset was used to develop QSAR models for predicting DILI. It should be noted that a total of 287 unique hepatotoxic compounds from CTD were retained. For external validation, test sets compiled by Ai et al. [37], Zhang et al. [49], and Kotsampasakou et al. [38] were adopted. Both the training set and external test sets used in this work were listed in Supplementary File 1.

### 2.2. Descriptors

As a freely available software package for calculating molecular descriptors and fingerprints, PaDEL-Descriptor [51] has been successfully used in many QSAR studies. In the current study, to characterize the molecular structural property of compounds, 12 types of fingerprints and a series of 2D molecular descriptors were generated by PaDEL-Descriptor (version 2.21) and detailed in Table 1. A detailed description focused on those molecular fingerprints/descriptors is provided in Supplementary File 1 (Fingerprints-descriptors).

### 2.3. Feature Selection

Feature selection is an effective method to eliminate redundant and irrelevant features. In the present work, we selected the features through two steps, as follows: Firstly, Boruta, an all-relevant feature selection wrapper algorithm, was adopted to select all features related to the hepatotoxic label [52]. As detailed in Table 1, for the 13 types of molecular fingerprints/descriptors, the number of molecular features identified by Boruta ranged from 13 to 138. Then, Pearson’s correlation analysis was conducted, focusing on those features. To remove the highly correlated features (Pearson’s correlation coefficients > 0.90), the findCorrelation function from the R package (version 3.5.3) caret was used. Finally, RFE from sklearn. feature_selection was implemented to rank the features. The parameter configuration of RFE was listed as follows: n_features_to_select = 1, step = 1. To perform RFE, a machine learning algorithm is required to assess the importance of each feature. Herein, we adopted the RandomForestClassifier from the sklearn. ensemble with the default parameter settings.

### 2.4. Machine Learning Models

In this work, all of the data mining tasks were implemented against Waikato Environment for Knowledge Analysis (WEKA, version 3.8.3) [53] within 10-fold cross validation. As an open and freely available machine learning tool, WEKA contains several different categories of supervised classifiers, such as bayes, functions, lazy, meta, and trees. To integrate the advantages of different categories of classifiers, we selected one or two representative algorithms in each category of classifier. As a result, a total of eight machine learning algorithms were selected, including NaiveBayes (bayes), LibSVM (function), IBK (lazy), KStar (lazy), AdaboostM1-J48 (meta), Bagging-IBK (meta), J48 (trees), and RandomForest (tree). Detailed descriptions of the basic theory of these machine learning algorithms are available in our previous paper [54].

**Figure 1:**
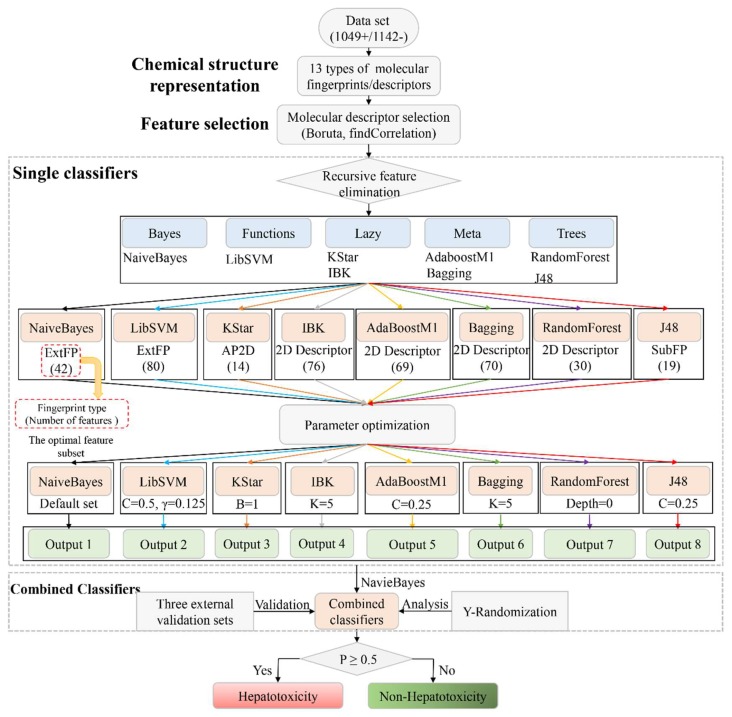
Workflow illustrating the combined classifier framework for predicting drug induced liver injury.

The detailed model-building workflow was displayed in Figure 1.

(1) Single classifiers: For each type of molecular fingerprint/descriptor, the non-redundant features listed in Table 1 were used as input to implement the eight machine learning algorithms selected.
  Then, RFE was performed to identify the optimal feature subset for each machine learning algorithm. We updated the feature subset of each type of molecular fingerprint/descriptor according to the feature rankings provided by the RFE method. We removed the lowest-ranked feature, and the remaining features were used as input to train the eight machine learning algorithms again. This step was repeated until the number of features was reduced to 1. As a result, a total of 677 models were developed for each machine learning algorithm. Then comparisons among those models were conducted, by which we attempted to identify the optimal feature subset for each machine learning algorithm. For each machine learning algorithm, the feature subset generating the highest average value of area under curve (AUC) and accuracy (ACC) was selected as the optimal feature subset. Of note, herein, all of the machine learning algorithms were implemented with the default parameter settings provided by WEKA. The optimal feature subsets for all of the eight machine learning algorithms are available in Table 3 and are detailed in Supplementary File 1 (Optimal feature subsets).
(2) Parameter optimization: In the development of the single classifiers, CVParameterSelection and GridSearch, two commonly used parameter optimization strategies, were adopted to determine the optimal parameter for each machine learning algorithm. The detailed parameter optimization information is provided in Table 2. All of the parameter optimization methods were implemented against WEKA within 10-fold cross validation.
(3) Combined classifiers: It has been proven that the combined classifiers strategy outperformed many classic classifier-fusion techniques (such as mean, maximum, and majority vote) [55]. In this work, the eight best single classifiers were utilized to predict the training sets. For each compound in the training set, eight predicted results (positive/negative) were provided by the eight best single classifiers. Then, those eight predicted results were defined as new descriptors and used as input to train NaiveBayes algorithm. As a result, a combined classifier focused on predicting DILI was developed.
(4) External Validation: By integrating the three external validation sets collected by Ai et al. [37], Zhang et al. [49], and Kotsampasakou et al. [38], an integrated external validation set was acquired and utilized to test the generalization ability of our model. In addition, to investigate the model’s robustness and statistical significance, Y-randomization analysis was performed [56].

### 2.5. Performance Metrics

To assess the predictive ability of the QSAR models, four parameters were calculated and defined as follows:

(1) $$\mathrm{Accuracy}=\frac{\left(\mathrm{True}\;\mathrm{positives}+\mathrm{True}\;\mathrm{negatives}\right)}{\left(N\right)}$$

(2) $$\mathrm{Specificity}=\frac{\mathrm{True}\;\mathrm{negatives}}{\left(\mathrm{True}\;\mathrm{negatives}+\mathrm{False}\;\mathrm{positives}\right)}$$

(3) $$\mathrm{Sensitivity}=\frac{\mathrm{True}\;\mathrm{positives}}{\left(\mathrm{True}\;\mathrm{positives}+\mathrm{False}\;\mathrm{negatives}\right)}$$

(4) $$\mathrm{Balanced}\;\mathrm{accuracy}=\frac{\left(\mathrm{Sensitivity}+\mathrm{Specificity}\right)}{2}$$

where N represents the size of the training set, and positives and negatives represent hepatotoxicants and non-hepatotoxicants, respectively.

In addition, AUC, another important indicator, was also calculated through the receiver operating characteristic analysis [57].

### 2.6. Herb-Hepatotoxic Ingredient Network

In our previous study, we collected hundreds of hepatotoxic ingredients and hepatotoxic herbs. Then, based on the ”herb-ingredient” pair data included in several typical TCM databases, we constructed a herb–hepatotoxic ingredient network. A total of 187 hepatotoxic herbs and 223 hepatotoxic ingredients were included in the network [48]. In the present study, this herb-hepatotoxic ingredient network was used to identify the hepatotoxic ingredients in TCMs.

## 3. Results

### 3.1. The Construction of In Silico Models for Predicting Drug-Induced Liver Injury

#### 3.1.1. Single Classifiers

**Figure 2:**
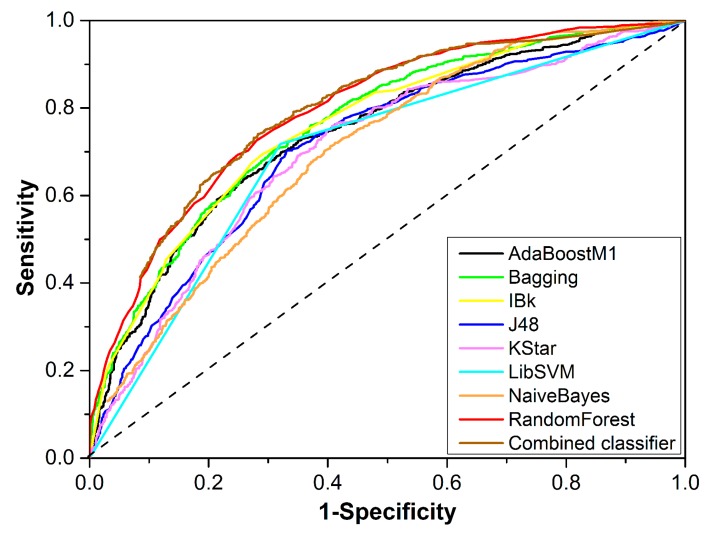
Receiver operating characteristic curves of the eight best single classifiers and the combined classifier.

The performance of the eight best single classifiers is provided in Table 3. The sizes of the optimal feature subsets ranged from 14 to 80. Four of the eight machine learning algorithms attained their best performance based on 2D Descriptor. In addition, single classifiers developed based on 2D Descriptor, including RandomForest, Bagging, AdaBoostM1, and IBk, showed greater AUC values than those developed based on molecular fingerprints, indicating the advantage of 2D Descriptor in the development of DILI prediction models. The highest AUC (0.794) was generated by RandomForest and 2D Descriptor when the feature number was reduced to 30. For that single classifier, ACC (accuracy), SE (sensitivity), SP (specificity), and BACC (balanced accuracy) were 72.250, 0.725, 0.720, and 0.723, respectively. For the other seven single classifiers, ACC varied between 64.993 and 70.196; AUC ranged from 0.699 to 0.762; SE, SP, and BACC were generally greater than 0.651, 0.625, and 0.651, respectively. Figure 2 provides the receiver-operating characteristic curves of the eight best single classifiers, displaying the classifiers’ performances more intuitively. In summary, most of the single classifiers did not provide a satisfactory performance, with their ACC values being less than 70%.

#### 3.1.2. Combined Classifier

By using the predicted results (positive/negative), of the eight best single classifiers developed in Section 3.1.1 as input, a combined classifier was built based on the NaiveBayes algorithm. The ACC, AUC, SE, SP, and BACC values of this combined classifier were 72.798, 0.793, 0.732, 0.724, and 0.728, respectively.

The combined classifier outperformed all the eight best single classifiers. For example, the ACC of the combined classifier was 72.798, which was higher than that of the eight best single classifiers by 0.548% to 7.805%. Among the eight best single classifiers, RandomForest classifier generated the highest ACC (72.250). Compared to RandomForest classifier, ACC, SE, SP, and BACC of the combined classifier improved by 0.548%, 0.700%, 0.400%, and 0.550%, respectively. For the external validation, by integrating the three external validation sets and eliminating the duplicates, we attained an integrated external validation set consisting of 125 positives and 79 negatives. Table 4 showed the performance of the combined classifier and the eight best single classifiers on the integrated external validation set. The combined classifier achieved the highest ACC (78.922), AUC (0.826), SP (0.750), and BACC (0.782), which further indicated that the combined classifier outperformed the eight best single classifiers. The predicted results of the integrated external validation set provided by the combined classifier are detailed in Supplementary File 1 (Integrated validation set). Summing up the above, the combined classifier outperformed the single classifiers both in cross validation and external validation, indicating the advantage and effectiveness of the combined classifiers strategy.

For further validation, 100 runs of Y-randomization were conducted. As a result, the ACC (50.8507 ± 0.9103) of the Y-randomization model was significantly worse than that of the combined classifier (ACC = 72.250), indicating the robustness and statistical significance of the combined classifier.

#### 3.1.3. Comparison to Prior Studies

Generally, performance comparisons to prior studies are indispensable. We compared our combined classifier to three, recently reported DILI prediction models from three aspects: sample size, cross validation, and external validation. Of note, for the external validation, the combined classifier was tested against the external validation sets collected by Ai et al. [36], Zhang et al. [42], and Kotsampasakou et al. [43] separately.

**Figure 3:**
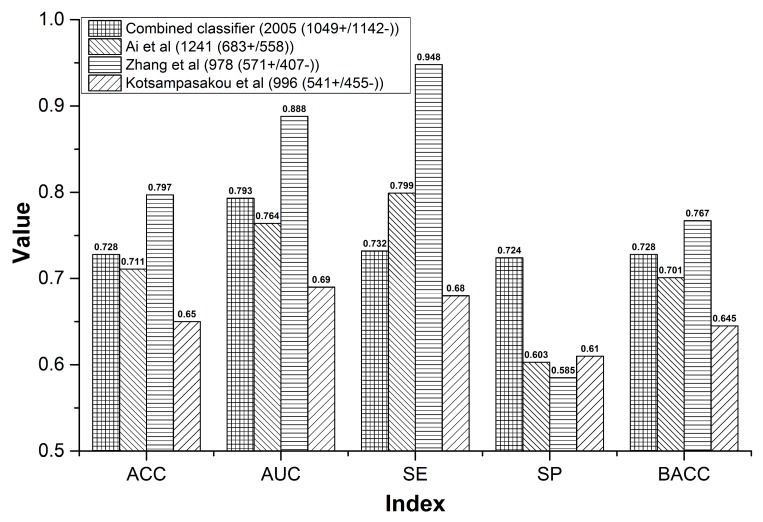
Comparisons between the combined classifier and prior studies within cross validation.

**Figure 4:**
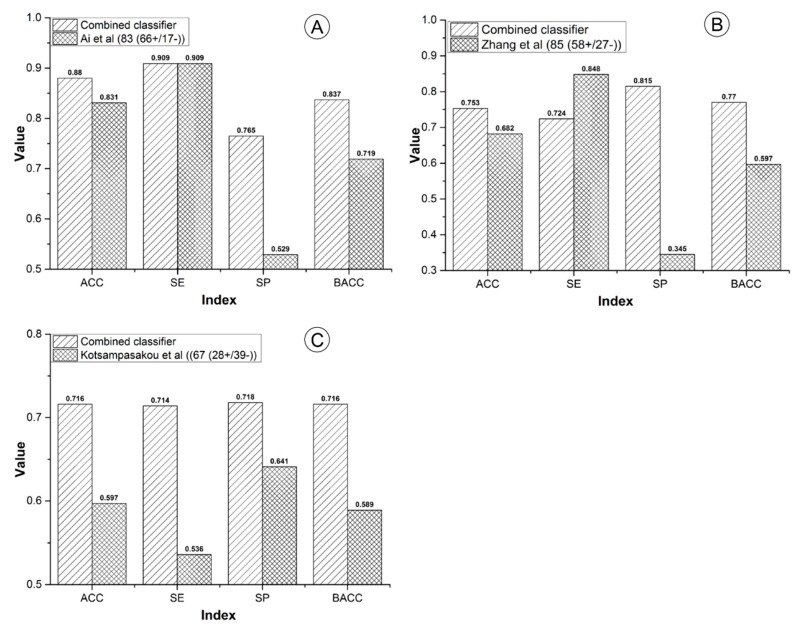
Comparisons between the combined classifier and prior studies on external validation sets. (**A**) The combined classifier versus Ai’s model; (**B**) the combined classifier versus Zhang’s model; (**C**) the combined classifier versus Kotsampasakou’s model.

For the sample size, our combined classifier and the other three models contained 2191, 1241, 978, and 996 compounds, respectively. The sample size of our combined classifier was 1.77 to 2.24 times of the other three models (Figure 3). Within cross validation, the SE of Ai’s model was 0.799, which was higher than that of our combined classifier by 6.7% (Figure 3). However, for the external validation, our combined classifier attained SE of 0.909, which is equal to that of Ai’s model (Figure 4A). In addition, both in cross validation (Figure 3) and external validation (Figure 4A), ACC, AUC, SP, and BACC of our combined classifier were significantly higher than that of Ai’s model. Within cross validation, Zhang’s model [48] attained the highest ACC, AUC, SE, and SP (Figure 3). However, SP of Zhang’s model was only 0.585, which reduced its application value significantly. Against Zhang’s external validation set, the ACC, SP, and BACC of our combined classifier were 0.753, 0.815, and 0.770, which are higher than those of Zhang’s model by 7.1%, 47%, and 17.3%, respectively (Figure 4B). The SE (0.848) of Zhang’s model was quite satisfactory. However, the SP was only 0.345. Zhang’s model provided very low SP value both in cross validation and external validation, which indicates that the results provided by Zhang’s model may contain a very number of false positives. In addition, all of the indexes provided by our combined classifier were significantly higher than those of Kotsampasakou’s model [38] both in cross validation (Figure 3) and external validation (Figure 4C).

Actually, compared to in silico models developed solely based on the synthetic drugs, it is more difficult for our combined classifier to attain a high accuracy. Both Zhao et al. [44] and Ye et al. [45] have demonstrated that models built solely based on synthetic drugs make it easier to attain a higher accuracy than those developed based on both TCMs and the synthetic drugs. The reason may be that the structures of natural products are more complex and diverse than those of synthetic drugs, increasing the difficulties of prediction.

In summary, the performance of our combined classifier was satisfactory. Both the cross validation and external validation indicated that our combined classifier was better than the prior studies. The detailed prediction results of the external validation sets collected by Ai et al. [36], Zhang et al. [42], and Kotsampasakou et al. [43] provided by the combined classifier and prior studies are available in Supplementary File 1 (Ai et al, Zhang et al, and Kotsampasakou et al).

### 3.2. A Computational Toxicology Approach to Screening the Hepatotoxic Ingredients in TCMs: Polygonum multiflorum Thunb as a Case

#### 3.2.1. The Computational Toxicology Approach

**Figure 5:**
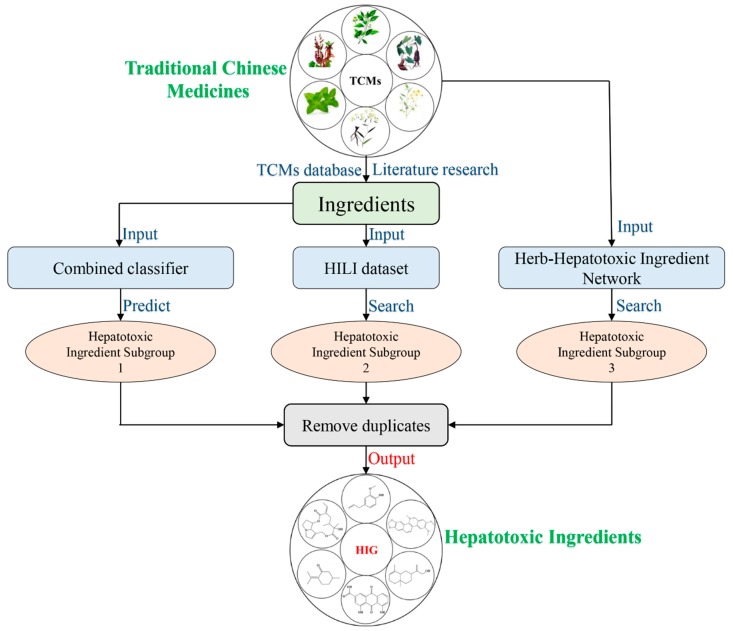
Diagram of the computational toxicology approach to identification the hepatotoxic ingredients in Traditional Chinese Medicines (TCMs).

In this section, we describe the computational toxicology approach proposed to screen the hepatotoxic ingredients in TCMs. The entire workflow of this computational toxicology approach is illustrated in Figure 5. The hepatotoxic ingredients of TCMs consisted of three hepatotoxic ingredient subgroups.

Hepatotoxic ingredient subgroup 1: The chemical ingredients of TCMs were extracted from TCM databases or collected from the literature. Then, the combined classifier developed in Section 3.1.2 was utilized to assess the hepatotoxic risk of each ingredient. The ingredients with higher hepatotoxic risks were marked as hepatotoxic ingredient subgroup 1.

Hepatotoxic ingredient subgroup 2: In our previous study, we developed a HILI dataset, which includes 296 hepatotoxic ingredients [48]. Herein, the chemical ingredients of TCMs were used as input to search this HILI dataset by name matching. The chemical ingredients of TCMs that appeared in the HILI dataset formed the hepatotoxic ingredients of subgroup 2.

Hepatotoxic ingredient subgroup 3: In our prior study, we constructed a herb-hepatotoxic ingredient network which includes 223 hepatotoxic ingredients and 187 hepatotoxic herbs [48]. Taking the herb name as input, one can obtain a hepatotoxic ingredient list of the corresponding herb. This hepatotoxic ingredient list was recorded as hepatotoxic ingredient subgroup 3.

Finally, the hepatotoxic ingredients were confirmed by integrating the three hepatotoxic ingredient subgroups and removing duplicates.

#### 3.2.2. *Polygonum multiflorum* Thunb as a Case

PmT, a commonly used herb in China, is known for blackening hair, tonifying the liver and kidney, and slowing the aging process. However, during recent decades, it has been reported to cause variable degrees of liver injury [58]. Taking PmT as an example, we attempted to investigate the reliability of the computational toxicity approach.

By retrieving three typical TCM databases, TCMSP, TCMID, and TCM Database@Taiwan, Wang et al. collected 98 chemical components of PmT [20]. Those chemical components were used as input to identify the hepatotoxic ingredients of PmT. All of those 98 ingredients were provided in Supplementary File 1 (PmT) where the hepatotoxic risk of each compound provided by our combined classifier was also available.

Hepatotoxic ingredient subgroup 1: Among the 98 chemical ingredients of PmT, 21 ingredients were predicted as hepatotoxic by our combined classifier. Those 21 ingredients formed the hepatotoxic ingredient subgroup 1 of PmT.

**Figure 6:**
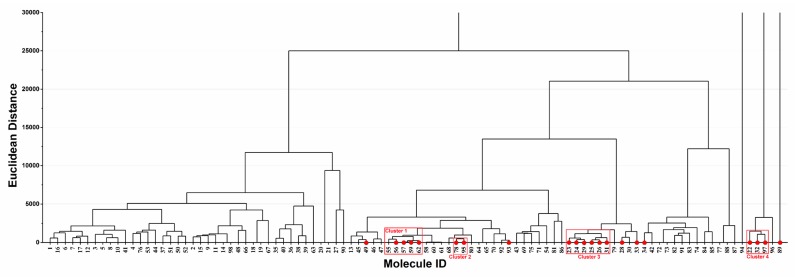
Hierarchical cluster analysis of the 98 ingredients in *Polygonum multiflorum* Thunb (PmT). The compounds predicted as hepatotoxicity by the combined classifier were highlighted with red solid circles. Molecule ID corresponds to ID in Supplementary File 1 (PmT).

Hierarchical cluster analysis based on Euclidean distance was performed to investigate the distribution of the 21 potential hepatotoxic compounds in the ingredient spectrum of PmT (Figure 6). As a result, the 21 hepatotoxic ingredients were significantly clustered into four major groups (Clusters 1–4). Ingredients in each cluster may have similar chemical characteristics. Such a cluster pattern was in line with the basic theory of QSAR, showing that our predicted results were reasonable to certain extent. In addition, consistency between the predicted results and reports in the literature for each compound was investigated and detailed in Supplementary File 1 (PmT). According to records in the literature, seven (Emodin, chrysophanol, rhein, danthron, aloe emodin, physcion, and Apigenin) out of the 21 hepatotoxic ingredients could cause variable degrees of liver injury. For the other 14 (Table 5 12–25) hepatotoxic ingredients, although direct evidence focused on their hepatotoxicity was not available, no one of them was reported to be potential hepatoprotector. A total of 77 chemical ingredients of PmT were predicted as non-hepatotoxicity, among which only three (Emodin 8-glucoside, physcion-8-O-D-glucopyranoside, and luteolin) chemical ingredients were reported to possess potential hepatotoxicity. For the other 74 chemical ingredients, no direct evidence about their hepatotoxicity was retrieved. Two (2,3,5,4’-tetrahydroxystilbene-2-O-β-D-glucopyranoside and resveratrol) of those 74 ingredients were reported to be non-hepatotoxic. In addition, a total of 17 ingredients among the 74 ingredients were reported to be potential hepatoprotectors. In summary, the predicted results of our combined classifier were highly consistent with reports in the literature, which further verified the reliability of the combined classifier.

Hepatotoxic ingredient subgroup 2: Taking the 98 chemical ingredients of PmT as input, we searched the HILI dataset by name matching. Resultantly, six ingredients (Table 5) were retrieved in the HILI dataset. Those six ingredients formed the hepatotoxic ingredient subgroup 2 of PmT.

Hepatotoxic ingredient subgroup 3: He Shou Wu, the pinyin name of PmT, was used as input to search the herb-hepatotoxic ingredient network. As a result, a total of seven hepatotoxic (Table 5) ingredients were searched, which formed the hepatotoxic ingredient subgroup 3 of PmT.

Finally, we acquired the hepatotoxic ingredients of PmT by integrating the three hepatotoxic ingredient subgroups of PmT and removing the duplicates. Table 5 shows the hepatotoxic ingredients of PmT, which consist of 25 compounds. The unique hepatotoxic ingredients from subgroup 1, subgroup 2, and subgroup 3 were 15, one, and three in number, respectively. The three hepatotoxic ingredient subgroups complemented each other, which showed the advantage of the computational toxicology approach.

## 4. Discussion

During the past few decades, a series of adverse effects caused by TCMs have been reported, among which hepatotoxicity is one of the major concerns. Identification of toxic ingredients is always considered to be the first step to illustrating the hepatotoxicity of TCMs. However, until now, an effective method to predict the hepatotoxicity of TCMs was unavailable. QSAR aims at finding the relationship between structure and activity/toxicity. In recent years, it has been widely used to predict the hepatotoxicity of the synthetic drugs. However, relevant research focused on TCMs is very rare. The major obstacle for such a situation is that dataset on the hepatotoxicity of TCMs is very limited. In our prior study, we collected a dataset on HILI, which laid the foundation for assessing the hepatotoxicity of TCMs based on the QSAR methods [48].

In this work, by utilizing eight machine learning algorithms and 13 types of molecular fingerprints/descriptors, a combined classifiers strategy was utilized to develop QSAR models for predicting the hepatotoxicity of TCMs. A total of 5416 single classifiers and one combined classifier were developed. For validation, an integrated external validation set was utilized to test the combined classifier. As a result, our combined classifier resulted in better performance than several prior studies both in cross validation and external validation. Within 10-fold cross validation, ACC of the combined classifier was 72.798, which was significantly higher than that of the Y-randomization model (ACC = 50.8507 ± 0.9103). The results mentioned above indicated that our combined classifier was stable and reliable. Another advantage of our combined classifier is the large scale of its training set. Our training set included 1049 positives and 1142 negatives. To the best of our knowledge, so large a DILI dataset is very rare. In addition, when predicting the hepatotoxicity of a compound, our combined classifier outputs the probability that the compound belongs to hepatotoxicants simultaneously. In this work, we set the threshold of the probability to 0.500. In other words, only those compounds with probabilities of hepatotoxicity greater than 0.500 would be categorized as hepatotoxicants, and all of the other compounds would be classified as non-hepatotoxicants. In practical applications, researchers can adjust the threshold of the probability according to their requirements. In our prior study, based on a training set consisting of 1254 compounds, we developed an in-silico model for predicting DILI by utilizing a voting method. Compared to our prior study, the advantages of this work include, but are not limited to, several aspects, as follows. Firstly, we built the combined classifier using 2191 unique compounds, 1.75 times more compounds than that used in our prior study. Secondly, a complex feature selection strategy was utilized to identify the optimal feature set for each machine learning algorithm. Thirdly, parameter optimization was performed in this work. Fourthly, the ACC, SE, SP, and BACC values of the combined classifier against the integrated external validation set were 78.922, 0.813, 0.750, and 0.782, which are higher than those of our prior work by 5.9%, 4.0%, 9.2%, and 6.6%, respectively.

**Figure 7:**
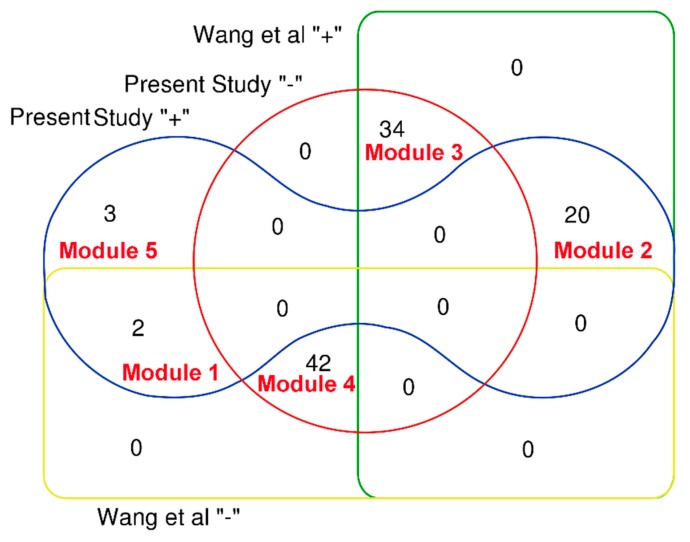
Venn diagram to show a comparison between the computational toxicology approach and the prior study. Prsent “+” and Prsent “−” indicate that the compound was predicted as hepatotoxic or non-hepatotoxic by our computational toxicology approach, respectively. Wang “+” and Wang “−” represent that the compound was identified as hepatotoxic or non-hepatotoxic by Wang et al, respectively. Ingredients included in each module are available in Supplementary File 1 (PmT).

Relying on a pathway-based systems toxicology approach, Wang et al. attempted to explore the hepatotoxicity of Pmt [20]. Firstly, the authors assessed the intestinal absorption properties of 98 compounds in PmT. Compounds with very poor intestinal absorption were discarded, and the remaining compounds were used for further analyses. Finally, a total of 44 compounds (Figure 7, Wang “−”) with very poor intestinal absorption were filtered off, and the other 54 compounds (Figure 7, Wang “+”) with good intestinal absorption were identified to be associated with the liver toxicity of PmT. In this work, taking PmT as a case, we proposed a computational toxicology approach to screen the heapotoxic ingredients in TCMs. The heapotoxic ingredients consisted of three heapotoxic ingredient subgroups which originated from the combined classifier, HILI dataset, and herb-hepatotoxic ingredient network, respectively. As a result, a total of 25 compounds in Pmt were identified as hepatotoxicants, among which 20 ingredients (Figure 7, module 2) exhibited good intestinal absorption. For the 44 compounds with very poor intestinal absorption, only 2 (Module 1 in Figure 7) of them were classified as hepatotoxicants. The most popular administration route of TCMs is oral administration. Therefore, chemical ingredients with good oral absorption are more likely to distribute in the liver and lead to liver injury. Therefore, we may speculate that screening focused on the oral absorption properties may help to narrow the range of screening hepatotoxic ingredients in TCMs. In introduction section, we highlighted that the potential hepatotoxicants identified by Wang et al. were the mixtures of toxic and beneficial ingredients. Thus, studies focused on differentiating hepatotoxicants and non-hepatotoxicants are required. In this work, for the 54 potential hepatotoxic compounds identified by Wang et al, twenty (Figure 7, module 2) of them were identified as hepatotoxicants by our computational toxicology approach. According to records in the literature, eight (Emodin, chrysophanol, rhein, danthron, aloe emodin, luteolin, physcion, and apigenin) out of those 20 hepatotoxic ingredients could cause varying degrees of liver injury. For the other 12 hepatotoxic ingredients, although direct evidence focused on their hepatotoxicity was not available, no one of them was reported to be a potential hepatoprotector. For the 34 non-hepatotoxic compounds (Figure 7, module 3), no direct evidence about their hepatotoxicity was retrieved. In addition, totals of 11 and one ingredients among these 34 non-hepatotoxic compounds were reported to be potential hepatoprotectors and a non-hepatotoxicant, respectively. Results provided by our integrated toxicity approach were highly consistent with reports in the literature. Summing up the above, we can conclude that the computational toxicity approach proposed in this work can differentiate non-hepatotoxic and hepatotoxic ingredients effectively. In addition, our computational toxicology approach also provided three (Chrysarobin, polygonumnolide C2, and emodin dianthrone) additional hepatotoxic ingredients in PmT which were not included in the 98 ingredients collected by Wang et al. Of note, among the 25 potential hepatotoxic ingredients in PmT, a total of 11 ingredients (Table 5, 1–11) have been reported to possess potential liver toxicity in the literature. For the other 14 ingredients, their hepatotoxicity has not been investigated. Therefore, experimental verification focused on the hepatotoxicity of these potential hepatotoxic ingredients is urgently needed in the future.

The major limitation of the study is that the majority of the HILI data used in this work was derived from animal/cell experiments rather than clinical case reports. The reason for such a situation is that studies on most ingredients from TCMs are still at the stage of animal-testing. Therefore, clinical data for these ingredients are generally unavailable. Nevertheless, we cannot deny the value of these data. After all, animal/cell experiments are often necessary in the discovery and development phases of novel drugs. Generally, researchers test new therapies through animal studies, and drug candidates which exhibit the most significant efficacy are moved on to clinical trials. Therefore, we must acknowledge the value of animal/cell experiments. Of course, if the HILI data used in this work were clinical, the value of this work would be greatly enhanced.

In summary, the advantages of this work can be summarized as, but not limited to, the following several aspects: Firstly, a large scale and high quality dataset for DILI was built, which will be a valuable resource for modeling/data mining in the future. Secondly, QSAR models developed solely based on the synthetic drugs often failed to assess the hepatotoxicity of natural products. Hundreds of hepatotoxic/non-hepatotoxic ingredients from medicinal plants were added into the modeling dataset, which led to our combined classifier, applicable to both natural products and synthetic drugs. Thirdly, the computational toxicology approach proposed in this work will assist in the screening of the hepatotoxic ingredients in TCMs, which will further lay the foundation for exploring the hepatotoxic mechanisms of TCMs. In addition, the method proposed in this work can be applied to research focused on other adverse effects of TCMs/synthetic drugs.

## 5. Conclusions

In the current study, by combining 13 types of molecular fingerprints/descriptors and eight machine learning algorithms, 5416 single classifiers were developed for predicting DILI. Then the NaiveBayes algorithm was utilized to construct a combined classifier by integrating the best single classifier of each machine learning algorithm. The combined classifier outperformed the eight best single classifiers and several prior studies in both cross validation and external validation. Subsequently, taking PmT as an example, a computational toxicology approach was proposed for the first time to uncover the hepatotoxic ingredients in TCMs. As a result, a total of 25 ingredients from PmT were identified as hepatotoxicants, among which 11 ingredients had been reported to be hepatotoxicants in the literature. The results were highly consistent with reports in the literature, indicating that our computational toxicology approach is reliable and effective in the screening of hepatotoxic ingredients in Pmt. In summary, the combined classifier can be used to assess the hepatotoxic risk of both natural compounds and synthetic drugs. The computational toxicity approach proposed in this work will be a powerful in silico methods for screening the hepatotoxic ingredients in TCMs.

## Figures and Tables

**Table 1:** Feature selection results of 13 types of molecular fingerprints/descriptors.

ID	Type	Number of Features		
		PaDEL-Descriptor	Boruta	Find Correlation
1	FP	1024	117	117
2	ExtFP	1024	111	111
3	EStateFP	79	13	12
4	GraphFP	1024	83	73
5	MACCSFP	166	59	52
6	PubchemFP	881	77	58
7	SubFP	307	25	22
8	SubFPC	307	18	15
9	KRFP	4860	61	49
10	KRFPC	4860	38	26
11	AP2D	780	38	34
12	APC2D	780	33	17
13	2D Descriptor	1444	138	91

**Table 2:** Parameter optimization.

Algorithm	Parameter	Parameter Optimization Method
RandomForest	**Depth**: The maximum depth of the tree	CVParameterSelection
Bagging	**K**: The number of neighbors to use	CVParameterSelection
AdaBoostM1	**C**: The confidence factor used for pruning	CVParameterSelection
IBk	**K**: The number of neighbors to use	CVParameterSelection
LibSVM	**C**: Penalty parameter, γ: The radial basis function kernel parameter	GridSearch
KStar	**B**: The parameter for global blending	CVParameterSelection
J48	**C**: The confidence factor used for pruning	CVParameterSelection

**Table 3:** Performance of the eight best single classifiers.

Classifiers	Type (Feature Number)	Parameter	ACC	AUC	SE	SP	BACC
RandomForest	2D Descriptor (30)	Depth = 0	72.250	0.794	0.725	0.720	0.723
Bagging	2D Descriptor (70)	K = 5	69.557	0.762	0.663	0.726	0.695
AdaBoostM1	2D Descriptor (69)	C = 0.25	68.736	0.743	0.651	0.721	0.686
IBk	2D Descriptor (76)	K = 5	70.196	0.758	0.673	0.729	0.701
LibSVM	ExtFP (80)	C = 0.5, γ = 0.125	69.786	0.699	0.718	0.680	0.699
KStar	AP2D (14)	B = 1	66.819	0.704	0.691	0.647	0.669
J48	SubFP (19)	C = 0.25	68.188	0.712	0.694	0.671	0.683
NaiveBayes	ExtFP (42)	Default set	64.993	0.704	0.677	0.625	0.651

**Table 4:** Performance of the eight best single classifiers and the combined classifier on the integrated external validation set.

Classifier	ACC	AUC	SE	SP	BACC
RandomForest	76.961	0.810	0.789	0.737	0.763
Bagging	69.118	0.764	0.711	0.658	0.685
AdaBoostM1	67.647	0.713	0.672	0.684	0.678
IBk	68.137	0.752	0.711	0.711	0.711
LibSVM	78.431	0.756	**0.867**	0.645	0.756
KStar	65.686	0.673	0.703	0.579	0.641
J48	64.216	0.632	0.711	0.526	0.619
NaiveBayes	61.275	0.603	0.719	0.434	0.577
Combined classifier	**78.922**	**0.826**	0.813	**0.750**	**0.782**

The maximum value of each index was highlighted with bold.

**Table 5:** Hepatotoxic ingredients in PmT identified by the computational toxicology approach.

ID	Ingredient	Liver Toxicity	Source		
			Subgroup 1	Subgroup 2	Subgroup 3
1	Emodin	[21]	+	+	+
2	Chrysophanol	[43]	+	+	+
3	Chrysarobin	[41]	none	none	**+**
4	Rhein	[44]	+	+	+
5	Danthron	[45]	+	none	+
6	Polygonumnolide C2	[41]	none	none	**+**
7	Emodin dianthrone	[41]	none	none	**+**
8	Aloe emodin	[46]	+	+	none
9	Luteolin	[26]	-	**+**	none
10	Physcion	[43]	+	+	none
11	Apigenin	[27]	**+**	none	none
12	Emodin-8-methyl ether	No report	**+**	none	none
13	Citreorosein	No report	**+**	none	none
14	Emodin-3-methyl ether	No report	**+**	none	none
15	Fallacinol	No report	**+**	none	none
16	2-Acetylemodin	No report	**+**	none	none
17	Hexadecanoic acid methyl ester	No report	**+**	none	none
18	Octadecanoic acid methyl ester	No report	**+**	none	none
19	Docosanoic acid methyl ester	No report	**+**	none	none
20	4-Hydroxybenzaldehyde	No report	**+**	none	none
21	2,5-dimethyl-7-hydroxychromone	No report	**+**	none	none
22	Hydroxymaltol	No report	**+**	none	none
23	Butanedioic acid	No report	**+**	none	none
24	Emodin-6,8-dimethylether	No report	**+**	none	none
25	Hexanoic acid	No report	**+**	none	none

“+” and “−” indicate hepatotoxic and non-hepatotoxic, respectively. The unique hepatotoxic ingredients from each subgroup are highlighted with red; “none” in column subgroup 1 means that the ingredient was not included in the 98 compounds of Pmt; “none” in subgroup 2 or subgroup 3 represents that the ingredient was not found in the HILI dataset or herb-hepatotoxic ingredient network, respectively.

## Supplementary Materials

Supplementary materials can be found at https://www.mdpi.com/2218-273X/9/10/577/s1. Supplementary File 1.

## Abbreviations

TCMs	Traditional Chinese Medicines
DILI	Drug-induced liver injury
PmT	*Polygonum multiflorum* Thunb
QSAR	Quantitative structure-activity relationship
CTD	Comparative Toxicogenomics Database
AUC	Area under curve
ACC	Accuracy
SE	Sensitivity
SP	Specificity
BACC	Balanced accuracy
HILI	Herb-induced liver injury
RFE	Recursive feature elimination
WEKA	Waikato Environment for Knowledge Analysis
